# Global Self-Organization of the Cellular Metabolic Structure

**DOI:** 10.1371/journal.pone.0003100

**Published:** 2008-08-29

**Authors:** Ildefonso M. De La Fuente, Luis Martínez, Alberto L. Pérez-Samartín, Leire Ormaetxea, Cristian Amezaga, Antonio Vera-López

**Affiliations:** 1 Departamento de Matemáticas, Facultad de Ciencia y Tecnología, Universidad del País Vasco, Vizcaya, Spain; 2 Departamento de Neurociencias, Facultad de Medicina, Universidad del País Vasco, Vizcaya, Spain; Tata Institute of Fundamental Research, India

## Abstract

**Background:**

Over many years, it has been assumed that enzymes work either in an isolated way, or organized in small catalytic groups. Several studies performed using “metabolic networks models” are helping to understand the degree of functional complexity that characterizes enzymatic dynamic systems. In a previous work, we used “dissipative metabolic networks” (DMNs) to show that enzymes can present a self-organized global functional structure, in which several sets of enzymes are always in an active state, whereas the rest of molecular catalytic sets exhibit dynamics of on-off changing states. We suggested that this kind of global metabolic dynamics might be a genuine and universal functional configuration of the cellular metabolic structure, common to all living cells. Later, a different group has shown experimentally that this kind of functional structure does, indeed, exist in several microorganisms.

**Methodology/Principal Findings:**

Here we have analyzed around 2.500.000 different DMNs in order to investigate the underlying mechanism of this dynamic global configuration. The numerical analyses that we have performed show that this global configuration is an emergent property inherent to the cellular metabolic dynamics. Concretely, we have found that the existence of a high number of enzymatic subsystems belonging to the DMNs is the fundamental element for the spontaneous emergence of a functional reactive structure characterized by a metabolic core formed by several sets of enzymes always in an active state.

**Conclusions/Significance:**

This self-organized dynamic structure seems to be an intrinsic characteristic of metabolism, common to all living cellular organisms. To better understand cellular functionality, it will be crucial to structurally characterize these enzymatic self-organized global structures.

## Introduction

One of the most important goals of contemporary biology is to understand the elemental principles governing metabolic structure as a whole which underlie the common design of all microorganisms and cells.

This global metabolic structure, conformed by the reactive interactions of thousands of biochemical species densely integrated through a labyrinthine web, represents one of the most complex dynamic systems in nature [1].

During the preceding two decades, different metabolic models have been studied intensively. Traditional models have focused on the kinetics of multi-enzyme systems by solving systems of differential equations and algebraic equations [2]. Petri's net theory, among others [3], has been applied to modelling metabolic pathways [4], decomposition of large metabolic networks into smaller subnetworks [5] and topological analysis of metabolic networks [6]. Large networks present many connections between the nodes, and their degree distributions follow a power law, so they can be considered as scale-free [7], [8]. The presence of “small-world” features [9] in scale-free networks is being studied [10], [11]. Constraint-based modeling approaches, such as flux-balance analysis, has been applied in several metabolic networks [12], [13]. Other mathematical models have been proposed to organize the networks both in its modular and hierarchical structure [14]–[16].

In an attempt to get a more accurate comprehension of the metabolic dynamic phenomena, we have proposed a dynamical system called “dissipative metabolic networks” or DMNs, which is basically formed by groups of enzymatic associations dissipatively structured and interconnected by fluxes and regulatory signals (allosteric and covalent).

Our model takes into account the fact that the cellular organization at the molecular level presents two relevant dynamic characteristics: the presence of enzymes aggregated in clusters and the emergence of dissipative catalytic patterns.

Experimental observations have shown that enzymes operate within metabolic pathways, may form functional catalytic associations and, thus, do not function in isolation of one another. Some of the first experimentally isolated enzymatic associations were, among others, the glycolytic subsystem [17], five enzymes from the cycle of the tricarboxylic acid [18], a triple multienzymatic-associate formed by the alpha-ketoglutarate dehydrogenase complex, the isocitrate dehydrogenase and the respiratory chain [19], and the complex formed by malate-dehydrogenase, fumarase and aspartate transferase [20]. Nowadays there are enough experimental data confirming the existence of numerous enzymatic associations belonging to metabolic routes, like lipid synthesis, glycolysis, protein synthesis, Krebs cycle, respiratory chain, purine synthesis, fatty acid oxidation, urea cycle, DNA and RNA synthesis, amino acid metabolism, cAMP degradation, etc. [21]–[28].

Association of various enzymes in large complexes (supramolecular organization) allows the direct transfer of their common intermediate metabolites (metabolic channelling). In addition, reversible interactions of enzymes with structural proteins and membranes are a common occurrence. This results in the existence of microcompartments within the soluble phases of cells. The microcompartmentation provides biophysical and biochemical mechanisms of physiological importance for the control of metabolic pathways [29]–[32].

The second consideration in our model is the presence of dissipative catalytic patterns. Each functional enzymatic association conforms a catalytic entity as a whole, in which spontaneously organized molecular oscillations may emerge.

In the *far from equilibrium* conditions prevailing inside the cell, the catalytic dynamics of enzymatic sets may present transitions between different stationary and oscillatory molecular patterns. When the enzymatic sets exhibit a rhythmic behavior, all the metabolic intermediaries oscillate with the same frequency but different amplitudes [33].

It is well known that these biochemical rhythms constitute a new type of supramolecular organization that may emerge in open systems far from equilibrium and was called dissipative structure by Prigogine [34].

It has been found experimentally that many enzymatic associations may cause oscillatory processes. The first systematic classification of cellular oscillatory behaviors gathered about 450 different kinds of biochemical rhythms [35], most of them corresponding to periodic oscillations [33], [36].

Nowadays, numerous experimental observations have contributed towards a better understanding of metabolic rhythms. For instance, it can be cited, among others: intracellular Ca^2+^ [37], free cytosolic ATP [38], intracellular pH [39], NAD(P)H [40], glycolysis [41], beta-oxidation of fatty acids [42], intracellular cAMP [43], metabolism of phospholipids [44], metabolism of amino acids [45], metabolism of mRNA [46], cellular respiratory processes [47], proteolysis [48], catalase reactions [49], Krebs cycle [50], and in protein kinase activities [51].

Likewise, numerous works have been carried out on the mathematical modelization of metabolic rhythms [52]–[59]. The understanding of the relation of metabolic rhythms to circadian processes is also a focus of notable interest [60], [61].

Due to the importance of cellular enzymatic rhythms, we have taken into account this aspect in our model of DMNs, so that the simulated catalytic processes can present both stationary and oscillatory activity regimes.

In the dissipatively structured enzymatic associations, the existence of allosteric enzymes permits the interconnection among them. The catalytic activity of the allosteric enzymes is modulated through the noncovalent binding of a specific metabolite at a place of the protein different from the catalytic site, provoking alterations of the metabolic state in an interval of seconds. Such types of modulation may be both positive (activation of their catalytic rates) and negative (inhibitory modulators). The regulation by means of the covalent interactions can originate “all-or-nothing” type answers [62].

In agreement with all these considerations, our DMNs model is composed of a set of catalytic elements (each of them represents a dissipatively structured enzymatic association called metabolic subsystem), which are connected by substrate fluxes and regulatory signals (allosteric and covalent modulations). The metabolic subsystems may present oscillatory and stationary activity patterns.

In a previous work [63], we have shown the rich variety of self-organised temporal patterns and global configurations that appears in the DMNs. One of them is the emergency of a functional configuration (similar to in vivo) in which a set of metabolic subsystems are always locked into active states (in eukaryotic cells they are mainly the Krebs cycle subsystem, the pyruvate dehydrogenase complex and the oxidative phosphorylation), whereas the rest of metabolic subsystems present dynamics of *on-off* changing states (glycolysis, beta-oxidation of fatty acids, amino acid degradations, gluconeogenesis, etc).

This type of functional metabolic structure has recently been shown by Almaas and colleagues for *Escherichia coli*, *Helicobacter pylori*, and *Saccharomyces cerevisiae* [12], [64].

In this paper, we have analyzed around 2.500.000 different metabolic networks in order to investigate the conditions in which this global configuration emerges. We have found that this functional metabolic structure is an emergent property of all dissipative metabolic networks with a high number of enzymatic subsystems.

## Results

### Model

Experimental observations have shown that the enzymes may form functional catalytic associations in which a new type of supramolecular self-organization may emerge (for more details, see the introduction section). These catalytic associations that operate within far from equilibrium conditions were called dissipative structures by Prigogine [34] and they conform a catalytic entity as a whole, in which the catalytic activity is autonomous with respect to the other enzymatic associations and molecular oscillations and steady states may emerge spontaneously. We have called metabolic subsystem to these groups of enzymatic associations dissipatively structured.

“Dissipative metabolic networks” (DMNs) are dynamical systems basically formed by a given number of groups of dissipatively structured enzymatic associations (called metabolic subsystems or MSbs) interconnected by fluxes of substrates and regulatory signals (both allosteric and covalent).

The metabolic subsystems receive distinct input fluxes of substrates and three possible types of regulatory signals: activatory, inhibitory (graduated) and total inhibitory (all-or nothing type). Each MSb transforms the input fluxes and regulatory signals into one or several output activities.

In agreement with experimental observations, the output activity of the MSbs may be oscillatory or steady state [33] and comprise an infinite number of distinct activity regimes.

In our model, when the set of dissipatively structured enzymes shows an activity with rhythmic behavior the output activities present nonlinear oscillations with different levels of complexity, as could be expected in the cellular conditions “in vivo”.

In order to be able to effect these different oscillatory or steady state patterns in each metabolic subsystem, the input-output conversion is made in two stages. First, the input fluxes are transformed into an intermediary activity by means of flux integration functions. In a second phase, the “intermediary activity” is modified by means of the “regulatory signals integration”, which depends on the combination of the received regulatory signals. Each regulatory signal has an associated regulatory coefficient which defines the intensity of its influence.

The parameters *q_i,j_*, called “influence coefficients”, that will be described in the materials and methods section, represent the influence of activatory and inhibitory modulators on the metabolic subsystems. These interactions correspond with the regulatory activity of the allosteric enzymes.

If the signal is of total inhibition (all-or nothing type), then we will use a parameter δ called threshold value (the level of the enzymatic covalent regulatory activity). When a given threshold value is reached it inhibits completely the activity of the MSb.

On the other hand, biological processes with long-term correlations are of notable interest in the study of complex dynamics.

Long-term correlations have been observed in, *e.g.*, the quantification of DNA patchiness [65], physiological time series [66], [67], NADPH series [68], DNA sequences [69]–[71], *K* ^+^ channel activity [72] and neural electrical activity [73], [74].

In a previous work with DMNs [63] we have analyzed different transitions generated by several metabolic networks and we have observed that these transitions exhibit long-term correlations (in the studied metabolic subsystems, their activity values depend to some extent on the previous ones).

In particular, we have characterized the behavior of several large complex transitions series generated in metabolic subsystems by means of the Hurst exponent H, which has been shown to be a robust and reliable test to detect the presence of long-term correlations [63], [74].

For a random series with independent increments, it can be shown that H should be equal to 0.5. If H≠0.5 this is indicative of the presence of “persistence” in the data, which means that the present state of the system is affected by previous states.

The studied time series generated by DMNs demonstrate complex transitions between the activities of the metabolic subsystems and we have found values for the Hurst exponent of 0.07<H<0.4, indicative of long term persistence over all the studied ranges. Values of H<0.5 are interpreted as characteristic of “trend-reversing” or “antipersistence” (a type of “persistence”). The behavior of the time series tends to reverse itself, for instance, a decreasing trend in the past usually implies an increasing trend in the future and conversely, an increase in the past is likely to be followed by a decrease. The high reliability of these results was tested with exhaustive Monte Carlo simulations. All the series studied presented persistence and these results showed clearly that the complex transitions in the DMNs exhibit long-term memory phenomena.

In an attempt to study a possible influence of the past activity on the enzymatic self-organized global structures we have considered the β parameter (the past influence coefficient) in some of the DMNs, in the sense that the present activity of each metabolic subsystem may be affected by its previous activities (see more details in the materials and methods section).

When the enzymatic activity is considered at the molecular level, the model must take into account the parameters for this level of organization. Thus, for example, we have studied the self-organization of certain enzymes at the molecular level; concretely, we have modelled the glycolytic subsystem by means of a system of differential equations with delay. In these studies, we have taken into account different molecular control parameters such as the Michaelis constant, dissociations constants, non-exclusive binding coefficients, equilibrium constants between conformational states, etc. [75]–[77].

On the other hand, experimentally, the dynamic structure of the cellular metabolism as a whole seems to be characterized by presenting a functional global configuration in which a metabolic core formed by several sets of enzymes are always in an active state, whereas the rest of the molecular catalytic sets exhibit dynamics of *on-off* changing states [12], [64].

Our main goal is to understand the cause for the emergence of this global supramolecular dynamic organization of enzymes. Since this main goal focuses on a superior level of organization than the molecular level, we will consider in this paper the emergent dynamic behaviors in the DMNs obtained by means of changes in: the level of enzymatic covalent regulatory activity, the level of allosteric activity, the number of metabolic subsystems, the flux topology, the topology of the regulatory signals and the flux values.

### Dissipative metabolic networks dynamics

Among the different dynamic behaviors that the DMNs may exhibit we have considered three aspects for our analyses.

1- Local activity developed by each metabolic subsystem (periodic and stationary patterns).
2- Dynamic transitions between steady states and periodic behaviors.
3- Global configurations that the DMNs may adopt:
  - All the subsystems are always in an *on* state.
  - All the metabolic subsystems always present cycles of activity-inactivity.
  - A certain number of subsystems are always locked in an *on* state conforming a metabolic core, while the rest of the subsystems are in an *on-off* switching state.

### Study of networks with two metabolic subsystems

**Figure 1:**
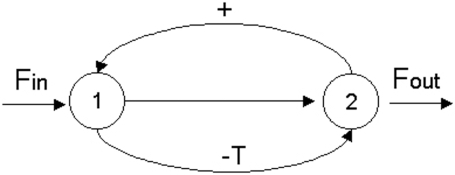
Network with two metabolic subsystems. DMN formed by two metabolic subsystems arranged in series with two feedback loops of regulatory signals (+, activator; −T, total inhibition). The MSb1 is activated by the second subsystem and the MSb2 is totally inhibited by the first subsystem when this one reaches a determinate threshold value.

In our first study, we have considered the simplest situation, which corresponds to a metabolic network formed only by two subsystems (concretely, we have analyzed the network described in the example of the materials and methods section and figure 1). In this study (see Table 1) the δ threshold value in the regulatory signals of total inhibition (the level of the enzymatic covalent regulatory activity) has been fixed as control parameter and we have taken the past influence coefficient β to be 0.

**Table 1:** Emergent dynamic behaviors as a function of δ in the DMN formed by two subsystems.

control parameter δ	MSb1	MSb2
0≤δ≤0.12	P_1_	Off
0.12<δ≤0.17	P_2_	Off
0.17<δ≤0.22	P_3_	On-Off SS_1_
0.22<δ≤0.25	P_6_	On-Off SS_1_
0.25<δ≤0.29	P_12_	On-Off SS_1_
0.29<δ≤0.37	P_8_	On-Off SS_1_
0.37<δ≤0.42	P_8_	On-Off SS-P
0.42<δ≤0.53	P_24_	On-Off SS-P
0.53<δ≤0.58	P_60_	On-Off SS-P
0.58<δ≤0.64	P_40_	On-Off SS-P
0.64<δ≤0.66	P_20_	On-Off SS-P
δ = 0.67	P_60_	On-Off SS-P
δ = 0.68	P_30_	On SS-P
0.68<δ≤0.77	P_12_	On SS-P
δ = 0.78	P_42_	On SS-P
0.78<δ≤0.82	P_56_	On SS-P
0.82<δ≤0.836	P_>100_	On SS-P_>100_
0.836<δ≤1	Chaos	On Chaos

*on*: the metabolic subsystem is always in an active state. *on-off*: the MSb always presents cycles of activity-inactivity. *off*: The metabolic subsystem always presents an inactive state. Pn: the output activity of the subsystem makes uninterrupted transitions between *n* different kinds of periodic oscillations and steady states. Chaos: the metabolic subsystem exhibits spontaneously infinite transitions between different behaviors oscillatory periodic and steady states. SS1: the metabolic subsystem presents a unique steady state. SS-P: the second subsystem presents cycles of activity-inactivity with different patterns of transitions between steady states and periodic behaviors. MSb1: metabolic subsystem 1. MSb2: metabolic subsystem 2. δ: the level of the enzymatic covalent regulatory activity (threshold value).

**Figure 2:**
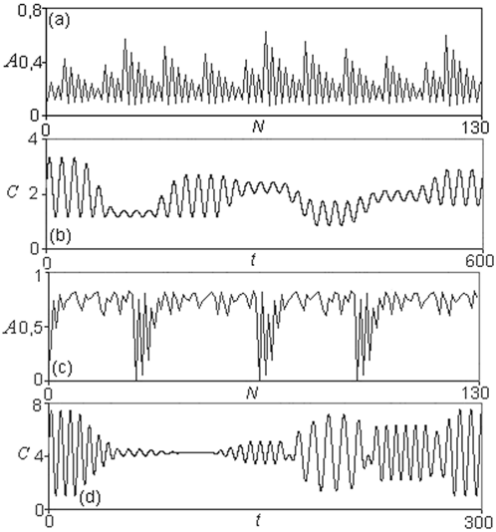
Dynamical patterns in the DMNs formed by two subsystems represented in the figure 1. (a) Periodic transitions in the mean amplitude A_0_ of the MSb1, and (b) their corresponding cycle of the different periodic behaviors belonging to the activity of the own metabolic subsystem; the δ threshold value is δ = 0.3, which represents the level of the covalent regulatory activity. (c) Complex periodic transitions in the mean amplitude A_0_ in the MSb2 for δ = 0.83 and (d) their corresponding patterns of its activity showing cycles of periodic oscillations with a steady state. The mean amplitude A_0_ is represented as a function of the number of transitions *N*. The activity C (sequences of periodic or stationary patterns) developed by each metabolic subsystem is represented as a function of the time *t*.

At small threshold values, for 0≤δ≤0.12 the MSb1 presents a single oscillatory behavior of one-period. In the interval 0.12<δ≤0.836 the first subsystem is always active and presents different cycles of transitions with 2, 3, 6, 12… and more of 100 periodic patterns. For instance, one can observe how for δ = 0.19 the MSb1, presents a cycle of period three, i.e. the output activity of the subsystem 1 makes uninterrupted transitions between three different kinds of periodic oscillations. In figure 2b, it can also be observed how, for δ = 0.3, a cycle of uninterrupted transitions between 8 different periodic oscillations emerges spontaneously in MSb1.

Finally, for 0.836<δ≤1 deterministic chaotic transitions can be observed. In this range of δ values, the network formed by only two metabolic subsystems spontaneously auto-organizes, provoking the emergence of a very complex chaotic behavior in which each subsystem presents infinite transitions between different periodic patterns. In this situation, the MSb1 modifies uninterruptedly its activity so that it never repeats itself for arbitrary long periods of time.

The MSb2 for 0≤δ≤0.17 is inactive (small threshold values δ represent high covalent regulatory activity). For 0.17<δ≤0.67 the second subsystem presents cycles of activity-inactivity with different patterns of transitions between steady states and periodic behaviors. For 0.68≤δ≤1 the MSb2 is locked in an active state. The same as with the first subsystem, for 0.837≤δ≤1 deterministic chaotic transitions between steady states and periodic behaviors can be observed, that is, the MSb2 spontaneously exhibits infinite transitions between different oscillatory periodic behaviors and steady states.

In figure 2, some dynamical transitions corresponding to the mean amplitude A_0_ and the corresponding periodic and stationary patterns developed by the two subsystems are shown.

The mechanism that determines these behaviors in both subsystems is not prefixed in any of the parts of the system. There is no feedback with oscillatory properties nor any other rules that determine the system to present complex transitions in the output activities of the metabolic subsystems. The dynamic behaviors which emerge spontaneously in the network have their origin in the regulatory structure of the feedback loops, and in the non-linearity of the constitutive equations of the system (see materials and methods section for more details).

The introduction in our analyses of a new parameter, the β past influence coefficient, allows us to observe some changes in the dynamic behaviors of the metabolic nets formed by only two subsystems (see Table 2).

**Table 2:** Emergent dynamic behaviors in function of β and δ in the DMN formed by two subsystems.

β	MSb1		MSb2			β	MSb1		MSb2		
1	0≤δ≤0.2	P	0≤δ≤0.2	-	Off	0.4	0≤δ≤0.1	P	0≤δ≤0.2	-	Off
	0.2<δ≤0.8	Ch	0.2<δ≤0.7	Ch	On-Off		0.1<δ≤0.7	Ch	0.2<δ≤0.7	Ch	On-Off
	0.8<δ≤1	P	0.7<δ≤1	-	Off		δ = 0.8	P	δ = 0.8	P	On
0.9	0≤δ≤0.2	P	0≤δ≤0.2	-	Off		0.8<δ≤1	Ch	0.8<δ≤1	Ch	On
	0.2<δ≤0.8	Ch	0.2<δ≤0.8	Ch	On-Off	0.3	0≤δ≤0.2	P	0≤δ≤0.2	-	Off
	0.8<δ≤1	P	0.8<δ≤1	P	On-Off		0.2<δ≤0.6	Ch	0.2<δ≤0.6	Ch	On-Off
0.8	0≤δ≤0.1	P	0≤δ≤0.2	-	Off		0.6<δ≤0.8	P	0.6<δ≤0.8	P	On
	0.1<δ≤1	Ch	0.2<δ≤1	Ch	On-Off		0.8<δ≤1	Ch	0.8<δ≤1	Ch	On
0.7	0≤δ≤0.1	P	0≤δ≤0.2	-	Off	0.2	0≤δ≤0.2	P	0≤δ≤0.2	-	Off
	0.1<δ≤1	Ch	0.2<δ≤0.8	Ch	On-Off		δ = 0.3	Ch	δ = 0.3	Ch	On-off
			0.8<δ≤1	Ch	On		0.3<δ≤0.7	P	0.3<δ≤0.6	P	On-Off
0.6	0≤δ≤0.1	P	0≤δ≤0.2	-	Off		0.7<δ≤1	Ch	δ = 0.7	P	On
	0.1<δ≤1	Ch	0.2<δ≤0.8	Ch	On-Off				0.7<δ≤1	Ch	On
			0.8<δ≤1	Ch	On	0.1	0≤δ≤0.8	P	0≤δ≤0.2	-	Off
0.5	0≤δ≤0.1	P	0≤δ≤0.2	-	Off		0.8<δ≤1	Ch	0.2<δ≤0.6	P	On-Off
	0.1<δ≤0.6	Ch	0.2<δ≤0.6	Ch	On-Off				0.6<δ≤0.8	P	On
	0.6<δ≤0.8	P	δ = 0.7	P	On-Off				0.8<δ≤0.1	Ch	On
	0.8<δ≤1	Ch	δ = 0.8	P	On						
			0.8<δ≤1	Ch	On						

β: the past influence coefficient. δ: the level of the enzymatic covalent regulatory activity (threshold value). Each metabolic subsystem may present one of the following three states: *on*: the metabolic subsystem is always in an active state, *on-off*: the MSb always present cycles of activity-inactivity, *off*: The metabolic subsystem always presents an inactive state. Ch: deterministic chaotic behaviors. P: transitions between periodic and/or stationary behaviors. MSb1: metabolic subsystem 1. MSb2: metabolic subsystem 2.

At small past influence coefficient β (β = 0.1), the threshold values δ do not provoke qualitative changes in the network. So, for 0≤δ≤1 the first subsystem is always *on* and chaos emerges between 0.8<δ≤1. In these conditions, the second subsystem exhibits three different activity states: for 0≤δ≤0.2 the MSb2 is inactive, for 0.2<δ≤0.6 its state is *on-off* and for 0.6<δ≤1 the second subsystem presents an *on* state. Chaos emerges when 0.8<δ≤1.

When β = 0.2 the MSb1 is always *on* and chaos emerges in two parametric regions: for δ = 0.3 and for 0.7<δ≤1. The second subsystem also presents, as the MSb1, chaotic behaviors under these conditions, however, for δ = 0.3 the MSb2 exhibits an *on-off* state, and when 0.7≤δ≤1 its state is *on*.

As β increases, the regions of chaotic behavior augment in correlation with δ. For instance, when β = 0.5 and either 0.1<δ≤0.6 or 0.8<δ≤1 chaos emerge in the metabolic net, the first subsystem is always *on* and MSb2 is *on-off* in the first range and is *on* in the second one.

If β = 1 the net presents only one chaotic parametric region. The first subsystem is always *on*, and chaotic transitions emerges when 0.2<δ≤0.8. The second subsystem exhibits two different behaviors: for 0≤δ≤0.2 and 0.7≤δ≤1 its state is *off*, and when 0.2<δ≤0.7 its state is *on-off*; as occurs in the MSb1, chaotic transitions between steady state and periodic behaviors emerges for 0.2<δ≤0.7.

If we take δ as the first reference parameter, it can be observed in Table 2 that, for relatively large values of β (0.6≤β≤0.8), the deterministic chaotic behaviors emerge for almost all the values of δ (the MSb1 for 0.1<δ≤1 and the MSb2 for 0.2<δ≤1). When β takes small values (0.2<β≤0.1) chaotic behaviors emerge for high values of δ.

On the other hand, it can be observed that the MSb1 is always active for all values of δ and β. However, the MSb2 may present one of three general states: always *on*, always *off*, or an *on-off* changing dynamics. Thus, for low values of δ (0≤δ≤0.2) the metabolic subsystem 2 is always inactive (*off*) independently of the values of β. For high values of δ the MSb2 shows a behavior in which it is always active (*on*) (0.6<δ≤1 for 0.3≤β≤0.1, 0.8≤δ≤1 for 0.4≤β≤0.5 and 0.8<δ≤1 for 0.6≤β≤0.7). Finally, the MSb2 presents dynamics of change active-inactive (*on-off*) for medium values of δ [0.3–0.6], with 0.2≤δ≤1 as the maximum range for β = 0.8.

### Study of metabolic networks with twelve metabolic subsystems

**Figure 3:**
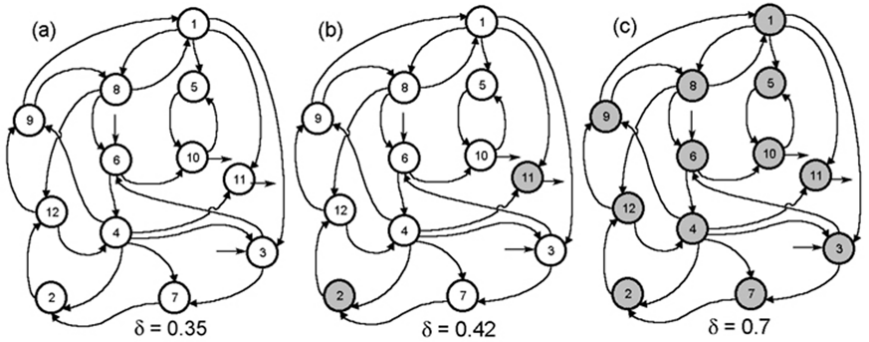
Global configurations in dissipative metabolic networks. In the DMN three kinds of global configurations may emerge. (a) All the subsystems exhibit an *on-off* changeable state, the δ threshold value parameter (the level of the covalent regulatory activity) is δ = 0.35. (b) A set of metabolic subsystems are locked into an active state (the MSb2 and the MSb11) while the rest of metabolic subsystems exhibit an *on-off* changeable state, for δ = 0.42. (c) The modification of the δ control parameter leads to a new global configuration of the network in which all the subsystems are in an *on* state, δ = 0.7. Arrows show substrate fluxes, the subsystems locked always in an active state are shown by dark circles and the metabolic subsystem that always present cycles of activity-inactivity are represented by white circles.

More interesting situations appear in the DMNs with more than one flux and one regulatory signal associated to each subsystem. In figure 3 we show a type of DMN formed by twelve subsystems (in the graphics only the interconnections by fluxes are reflected; the corresponding integration function parameters and the coefficient values of the regulatory signals are shown in Table 3).

**Table 3:** Coefficient values of the regulatory signals and integration function parameters belonging to the DMN formed by 12 subsystems.

MSb	Fluses in		Flux Parameter 1°			Flux Parameter 2°			Initial Conditions		
1	9	8	.88	.80	.95	.78	.53	.74	.53	.16	.75
2	7	4	.93	.87	.99	.61	.53	.67	.97	.27	.48
3	4	1	.96	.74	.69	.90	.85	.88	.79	.80	.65
4	6	12	.88	.75	.52	.50	.68	.51	.73	.13	.59
5	10	1	.95	.73	.67	.97	.78	.73	.17	.19	.27
6	3	8	.58	.96	.53	.80	.60	.64	.19	.28	.48
7	3	4	.81	.79	.61	.85	.77	.95	.05	.66	.68
8	9	1	.91	.51	.51	.91	.68	.57	.32	.52	.54
9	12	4	.77	.99	.69	.62	.54	.63	.04	.21	.87
10	5	6	.98	.95	.96	.60	.73	.81	.37	.61	.78
11	4	1	.76	.98	.63	.51	.86	.55	.31	.77	.41
12	8	2	.52	.83	.76	.68	.94	.54	.34	.42	.17

MSb	Reg. Signals			Reg. Sign. Coef.			Reg. Sign. Coef.			Reg. Sign.Coef.		
1	10T	6T	2T	0	0	0	0	0	0	0	0	0
2	1−	14−	11−	.07	.82	.03	.19	.61	.36	.04	.77	.52
3	9+	5+	1T	.18	.43	.01	.14	.53	.47	0	0	0
4	6+	9T	12−	.19	.86	.29	0	0	0	.19	.58	.77
5	9−	8−	7T	.16	.43	.93	.009	.61	.17	0	0	0
6	1−	4−	2+	.06	.44	.76	.18	.95	.58	.02	.23	.34
7	4+	3T	1−	.1	.19	.66	0	0	0	.05	.43	.52
8	11+	7T	6T	.05	.68	.76	0	0	0	0	0	0
9	4−	6T	2+	.07	.32	.29	0	0	0	.18	.45	.76
10	5T	2T	1T	0	0	0	0	0	0	0	0	0
11	10−	2T	7+	.18	.63	.4	0	0	0	.05	.82	.01
12	9T	5+	2+	0	0	0	0.05	.002	.74	.02	.67	.09

Each subsystem may present three states: always *on*, always *off* and an *on-off* changing dynamic. If we take into account the dynamics followed by these subsystems, interesting functional configurations in the whole net can be observed.

Thus, when 0≤δ≤0.29 all the subsystems are inactive. For 0.29<δ≤0.39, all the subsystems are locked into an *on-off* changeable state (figure 3a). In the network a qualitative change in the dynamical structure emerges for 0.39<δ<0.68: various dissipative catalytic elements develop a set of subsystems locked in an *on* state, while the rest of metabolic subsystems are in an *on-off* changeable state (figure 3b). The modification of the control parameter in the range of 0.68≤δ≤1, leads to a new phase transition in which all the subsystems are in an *on* state (figure 3c).

In Table 4 are represented different global configurations in DMNs formed by twelve subsystems in function of β and δ. It is shown in bold the set of nets that present a self-organized global functional structure in which several subsets of enzymes are always in an active state (metabolic core) whereas the rest of molecular catalytic sets exhibit dynamics of *on-off* changing states. For very high values of β (0.9≤β≤1) never emerge DMNs characterized by presenting a metabolic core.

**Table 4:** Global functional configurations in the nets formed by twelve subsystems.

	β = 0	β = 0.1	β = 0.2	β = 0.3	β = 0.4	β = 0.5	β = 0.6	β = 0.7	β = 0.8	β = 0.9
δ = 0.1	10 On-Off	10 On-Off	10 On-Off	10 On-Off	10 On-Off	10 On-Off	10 On-Off	12 Off	12 Off	12 Off
	2 Off	2 Off	2 Off	2 Off	2 Off	2 Off	2 Off			
δ = 0.2	11 On-Off	11 On-Off	11 On-Off	11 On-Off	11 On-Off	12 On-Off	11 On-Off	12 Off	12 Off	12 Off
	1 Off	1 Off	1 Off	1 Off	1 Off		1 Off			
δ = 0.3	12 On-Off	11 On-Off	**11 On-Off**	12 On-Off	11 On-Off	12 On-Off	12 Off	12 Off	11 On-Off	12 Off
		1 Off	**1 On**		1 Off				1 Off	
δ = 0.4	**11 On-Off**	12 Off	**11 On-Off**	12 On-Off	12 On-Off	12 On-Off	12 On-Off	12 On-Off	12 On-Off	12 Off
	**1 On**		**1 On**							
δ = 0.5	**9 On-Off**	**9 On-Off**	**9 On-Off**	**9 On-Off**	12 On-Off	12 On-Off	12 On-Off	12 Off	12 Off	12 Off
	**3 On**	**3 On**	**3 On**	**2 On**						
δ = 0.6	**3 On-Off**	**9 On-Off**	**9 On-Off**	**9 On-Off**	**9 On-Off**	**9 On-Off**	12 On-Off	12 On- Off	12 On-Off	12 Off
	**9 On**	**3 On**	**3 On**	**3 On**	**3 On**	**3 On**				
δ = 0.7	12 On	12 On	12 On	**9 On-Off**	**9 On-Off**	**9 On-Off**	**11 On-Off**	12 On-Off	12 Off	12 Off
				**3 On**	**3 On**	**3 On**	**1 On**			
δ = 0.8	12 On	12 On	12 On	12 On	12 On	**5 On-Off**	12 On-Off	**11 On-Off**	2 On-Off	12 Off
						**7 On**		**1 On**	10 Off	
δ = 0.9	12 On	12 On	12 On	12 On	12 On	**8 On-Off**	**11 On-Off**	12 On-Off	2 On-Off	2 On-Off
						**4 On**	**1 On**		10 Off	10 Off
δ = 1	12 On	12 On	12 On	12 On	12 On	**4 On-Off**	**5 On-Off**	**5 On-Off**	**5 On-Off**	2 On-Off
						**8 On**	**7 On**	**7 On**	**7 On**	10 Off

In the DMNs different functional reactive structures may emerge spontaneously: all the subsystems are always in an *on* state, all the subsystems are always in an *on-off* changeable state, a certain number of metabolic subsystems are always locked in an *on* active state (metabolic core) while the rest of the subsystems remain in an *on-off* changing dynamics and nets in which all their subsystems are always *off* (nets functionally non-viable). It is shown in bold the set of nets in which a metabolic core emerges. β: the past influence coefficient. δ: the level of the enzymatic covalent regulatory activity (threshold value).

It can be observed also that in this kind of networks, for high covalent regulatory activity (0.1≤δ≤0.4) nets with all metabolic subsystems in an *on-off* changing dynamic emerge (small threshold values δ represent high covalent regulatory activity). Last, the DMNs with low levels of β and high values of δ present all their metabolic subsystems always active (*on*).

In order to better understand the influence of the control parameters δ and β on the global self-organizations in the DMNs, we have made a first study of different nets constituted by twelve metabolic subsystems with three regulatory signals and two input fluxes by subsystem. In this study all networks present the same flux configuration described in figure 3. The rest of parameters that configure the metabolic net, such as flux integration parameter, influence coefficients, and the regulatory interconnections were randomly chosen assigning the same probability to each type of regulatory signal.

**Figure 4:**
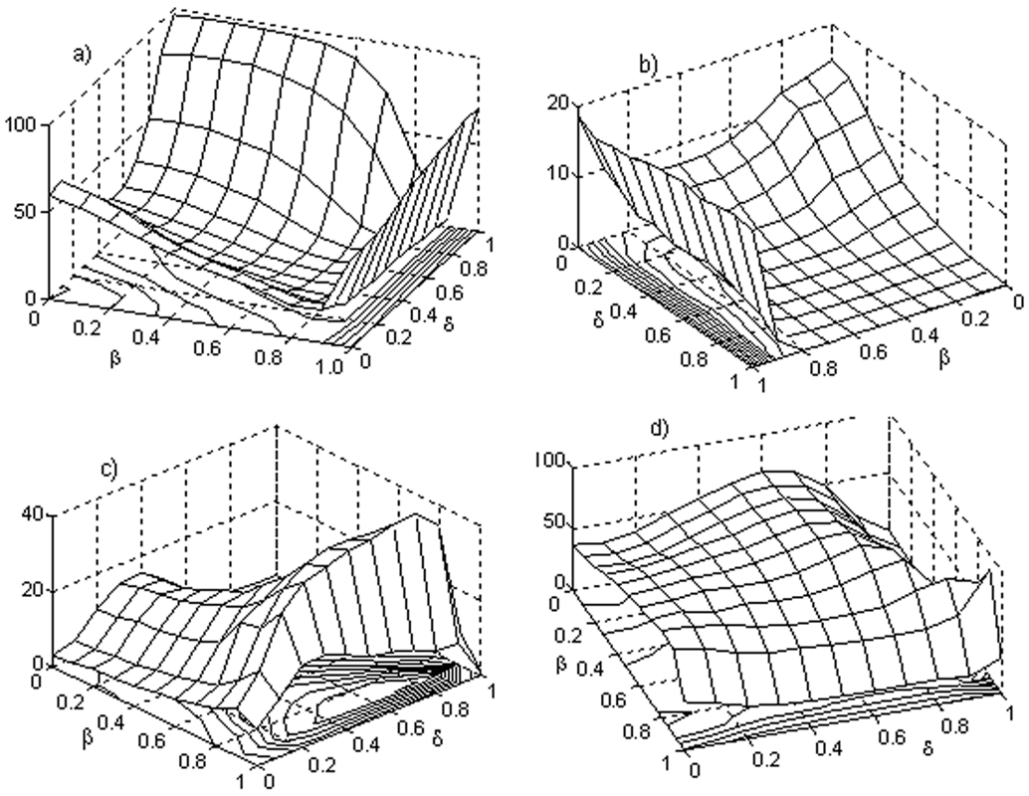
Analysis of DMN with 12 subsystems. Percentage of DMN that present, respectively, (a) all their subsystems unable to change the state (each subsystem is always *on* or is always *off* and never is in an *on-off* changeable state), (b) all subsystems in an *off* state (they constitute a particular case of the dissipative networks showed in figure 6a and they are functionally unviable metabolic nets), (c) all their elements in an *on-off* regime and (d) a subset of dissipative metabolic subsystems locked into an active state while the rest exhibit an *on-off* changeable state. In the horizontal axes the δ threshold value (the level of the covalent regulatory activity) and the β past influence coefficient are displayed. In total, 1.210.000 different randomly constructed metabolic nets with 12 subsystems and two input flux by subsystem were studied.

A total of 1.210.000 different metabolic nets were built, the results of this study are shown in figure 4. In the calculations, 10,000 different metabolic networks were taken into account for each represented point (corresponding to determinate values of δ and β), with 300 iterations per net. In these networks, for a given set of subsystems, random regulatory signals were settled. Each one of the regulatory feedbacks that connect the subsystems were randomly decided, and it was also decided randomly the type of regulation (activation, inhibition or total inhibition) as well as the parameters associated to each regulatory signal (see the materials and methods section for more details). The set of subsystems from whom a given subsystem receives fluxes was also decided randomly, (being thus established randomly the flux architecture of the net) as well as the parameters associated to the flux integration functions. The probability distribution that we used in the random selections was the uniform distribution. The control parameters were varied in steps of 0.1 in the unit interval [0, 1] and the criterion followed to determinate if a metabolic subsystem was *on*, *off* or changing *on-off* was that the subsystem had the corresponding state between the iterations 200 and 300.

In figure 4a, the percentage of DMNs with all their subsystems unable to change the state is shown (each subsystem is always *on* or is always *off* and never is in an *on-off* changeable state). It can be observed that if β is maximum (β = 1) all the percentages are greater than 50% (between 51.11% for δ = 0 and 70.66% for δ = 0.9). When the covalent regulation is very low (0.9<δ≤1) and 0<β≤0.6, the biggest percentages are reached (between 64% and 97.6%). For 0.3<δ≤0.7 and 0.6<β≤0.9 the lowest percentages are reached (all them are less than 10%).

The maximum percentage of dissipative networks with all its metabolic subsystems unable to change the state (97.6%) is obtained when δ = 1 and β = 0.1, and the minimum (1.39%) is attained for δ = 0.6 and β = 0.7.

In figure 4b the percentage of networks with all their subsystems in an *off* state is represented. They constitute a particular case of the DMNs showed in figure 4a and they are functionally unviable metabolic nets. When the past influence coefficient is maximum (β = 1) the biggest percentages are reached. These values range from 17.16% for δ = 0.1 to 19.97% for δ = 0.9. If 0.7≤δ≤1 and β≤0.7 all the percentages are less than 1%.

In figure 4c, the percentage of metabolic networks that present all their elements in an *on-off* regime is shown. The biggest percentages are obtained when β = 0.9 and 0.3≤δ≤0.9. The lowest percentages (always less than 1%) are found for a value of δ around 0.7 and β≤0.4. The maximum percentage (45.5%) is obtained when β = 0.9 and δ = 0.8, and the minimum (0.001%) is obtained when β = 0.4 and δ = 1.

Finally, figure 4d shows the percentage of nets characterized by having a subset of dissipative metabolic elements locked into an active state (metabolic core) while the rest exhibit an *on-off* changeable state. If the covalent regulatory activity is minimum (δ = 1), the percentage of nets presenting these functional configuration varies strongly as a function of β, in fact, the absolute maximum is 87.33% (β = 0.9) and the absolute minimum is 2.39% (β = 0.1). For a maximum covalent regulation (δ = 0) the biggest percentage is 71.37% (β = 0.8) and the minimum percentage is 37.09% (β = 0). When 0<δ<1, the highest percentages (about 80%) are attained for a given value of β between β = 0.5 and β = 0.7 and the minimum values correspond to β = 1. The 64.4% of the pairs (β, δ) present a percentage over 50% for these kind of metabolic nets.

A total of 33.26% of the studied DMNs correspond to the case in which all its subsystems are unable to change the state, the 14.09% of the nets present all their elements in an *on-off* regime, and the 52.56% are metabolic nets characterized by having a subset of dissipative metabolic elements locked into an active state while the rest exhibit an *on-off* changeable state. The percentage of networks with all their subsystems in an *off* state (functionally unviable metabolic nets) is 4.95% (this percentage constitutes part of the 33.26% of the nets unable to change its state).

It can be observed that the global configuration characterized by having a metabolic core is the one that presents the maximum percentage when compared with the other two active global configurations.

In metabolic networks formed by twelve subsystems, complex dynamical transitions in the activities of each MSb are common and we have observed that chaotic transitions between steady states and periodic behaviors may emerge both in subsystems always *on* (for example, when δ = 0.7 and β = 0) and in metabolic subsystems that exhibit an *on-off* changeable state (for example, when δ = 0.8 and β = 0.3).

### Chaos in dissipative metabolic networks

Under experimental conditions, most of the local dynamic behaviors of the metabolic subsystems present periodical oscillatory patterns or steady states [33]. However, under cellular conditions it has been observed that some subsystems can present chaotic local activities.

**Figure 5:**
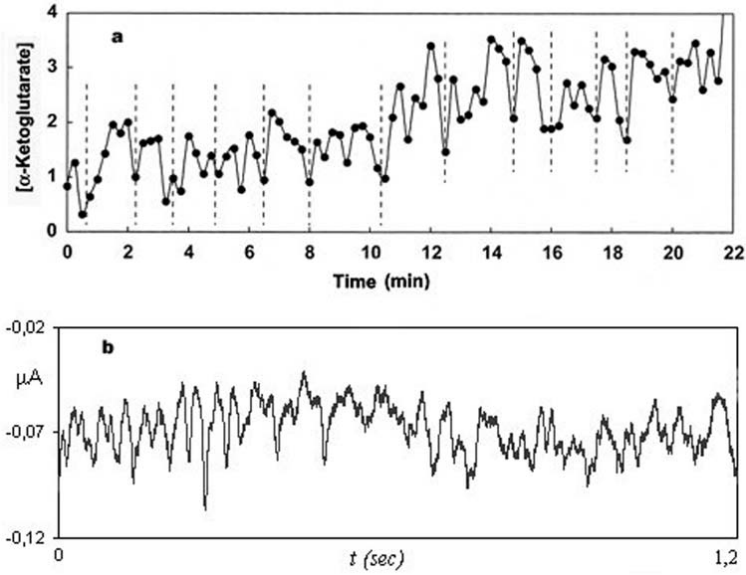
Chaotic behaviors in two different metabolic subsystems. (a) Experimental oscillations belonging to the citric acid cycle, a metabolic subsystem always active under cellular conditions (MacDonald et all, 2003), the α-ketoglutarate concentration is represented as a function of the time *t*. (b) Experimental calcium oscillations in oocytes carried out by our group. Application of Fetal Bovine Serum (FBS) (Sigma-Aldrich) diluted 1∶1000 to a *Xenopus laevis* oocyte promotes the generation of inward Ca^2+^-dependent Cl^−^ oscillating currents. The trace shown here corresponds to a stable phase of the whole current. These chaotic oscillations represent the activity of a subsystem in an active-inactive changeable regime. The amplitude (μA) is represented versus time.

In figure 5, two experimental chaotic behaviors are shown. The first one (figure 5a) corresponds to oscillations in citric acid cycle, a metabolic subsystem always active under cellular conditions [78] and the second (figure 5b) represents experimental calcium oscillations in *Xenopus laevis* oocyte carried out by our group. These chaotic oscillations correspond to a subsystem in an *on-off* changeable regime.

The blood serum from many animals contains a factor which activates a membrane receptor that is coupled to the phosphatidylinositol second messenger system and produces oscillatory currents. These currents are elicited by activation of *Cl* ^−^ channels sensitive to the intracellular 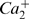 concentration [79].

The enzymatic activity bound to the membrane of the oocyte after the external stimulus of the Fetal Bovine Serum causes the dynamic mobilization of the intracellular calcium. This activity ceases soon after the exposure of the cell to the external agent.

**Figure 6:**
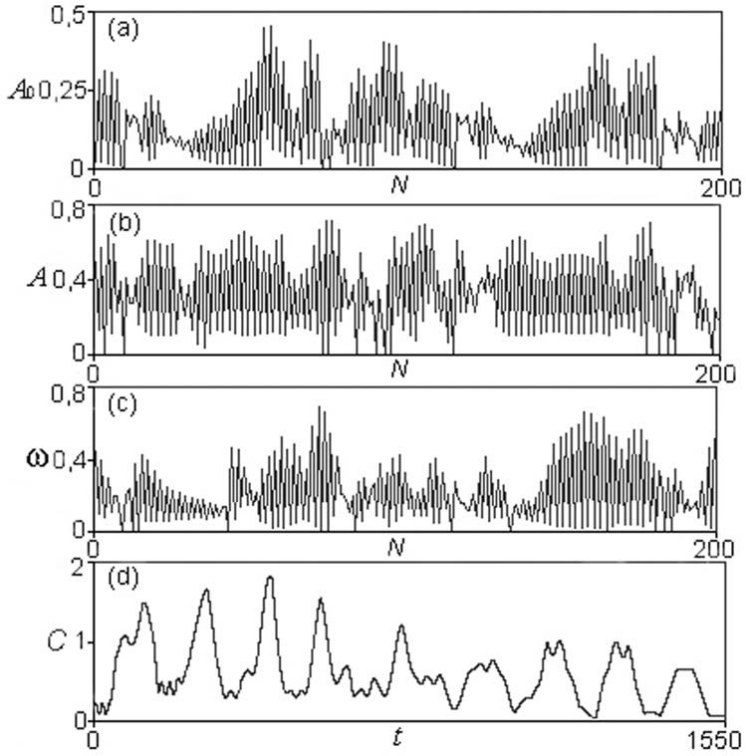
Chaotic behaviors in DMN. Chaotic time series generated by the sixth metabolic subsystem belonging to the network formed by twelve subsystems (figure 3). The control parameter values are δ = 0.8 and β = 0.3. Under these conditions the subsystem exhibits an *on-off* changeable state and it can be observed that the mean amplitude A_0_ (a), the amplitude *A* (b) and the frequency *ω* (c) present chaotic behaviors. In agreement with this kind of transitions the subsystem exhibits a metabolic activity pattern characterized by very complex transitions (d). The activity C of the metabolic subsystem, which represents the concentration of a determinate intermediate metabolite, is represented as a function of the time *t*.

Figure 6 shows time series generated by the MSb6 (belonging to the network formed by twelve subsystems) for δ = 0.8 and β = 0.3. Under these conditions the subsystem exhibits an *on-off* switching state and both the mean amplitude A_0_ (figure 6a), the amplitude *A* (figure 6b) and frequency *ω* (figure 6c) present chaotic behaviors. In agreement with this kind of transitions the subsystem (MSb6) exhibits metabolic activity patterns characterized by very complex oscillatory behaviors (figure 6d).

### Study of metabolic networks formed by high numbers of metabolic subsystems

**Figure 7:**
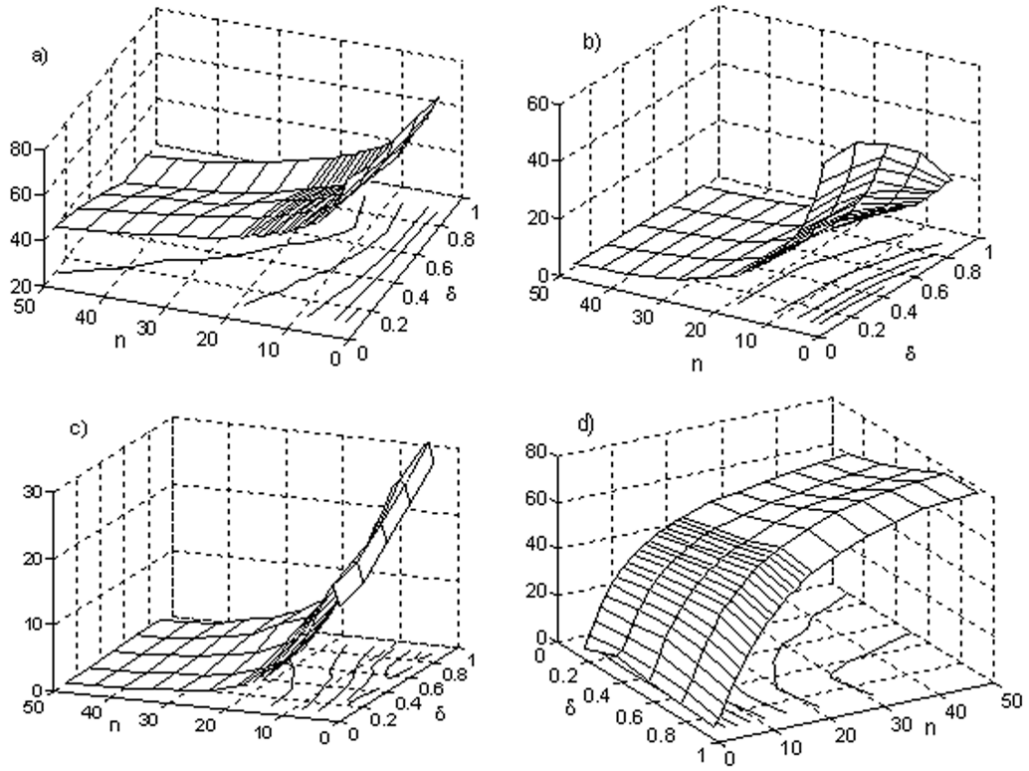
Analysis of DMN with a variable number of subsystems. Percentage of DMNs that exhibit, respectively, (a) all subsystems unable to change the state, (b) all subsystems in an *off* state (functionally unviable metabolic nets), (c) all their elements in an *on-off* regime and (d) a subset of dissipative metabolic subsystems locked into an active state while the rest exhibit an *on-off* changeable state. In the horizontal axes the δ threshold value (the level of the covalent regulatory activity) and the number of subsystems *n* are shown. In total, 1.250.000 randomly constructed nets were studied.

Finally, in order to study the behavior of the DMNs depending on δ (covalent regulation level) and *n* (number of subsystems), similar statistics were also carried out. In this case, networks formed by subsystems with one regulatory signal for each metabolic subsystem and one input flux were considered; likewise the same probability for each regulatory signal was assigned. Each net subsystem can have an outer input flux with a probability of 0.1, so that a metabolic network could have more than one outer input flux but at most one for each subsystem. Again, all parameters, the topology of the regulatory signals and flux interconnections (flux topology) were randomly configured. The β parameter was always taken equal to 0.3. For this study (figure 7) 1.250.000 different metabolic networks were analyzed.

In figure 7a, the percentage of metabolic nets with all their subsystems unable to change their state is shown. In this kind of nets, each subsystem is always *on* or is always *off*, and never is in an *on-off* switching state. It can be observed that for a fixed δ there is a remarkable trend towards a decrease in this percentage when the number of subsystems that conforms the net is increased. When *n = 2*, the mean percentage with respect to δ is 75.5% and for *n = 50*, it is 29.6%. With respect to the behavior of percentages when the number *n* of subsystems is fixed, two different clusters are observed.

For *n* up to 35 subsystems, the percentage decreases slightly with δ when δ is less than 0.8, and increases starting from this value. In this cluster, the maximum percentage (83.31%) is obtained when δ = 0.1 and *n = 2*, and the minimum percentage (24.81%) is obtained when δ = 0.7 and *n = 35*.

For a number of metabolic subsystems greater than 35, the percentage decreases steadily with respect to δ; this decreasing is very remarked for δ>0.5 (that is, when the covalent regulation is low, the percentage of nets with all their subsystems unable to change their state diminishes notably). In this second cluster, the maximum percentage (41.92%) is obtained when δ = 0.1 and *n = 40*, and the minimum percentage (21.65%) is obtained when δ = 0.9 and *n = 50*.

The DMNs in which all subsystems are off constitute a particular case of the nets in which all the metabolic subsystems are unable to change their state (figure 7b). It is observed that, when δ is fixed, the percentage goes asymptotically to zero. For a given value of n, the percentage decreases slowly with the increment of the δ value. The maximum percentage (56.37%) is obtained when *n = 2*, and its minimum (0.32%) is reached when *n = 50*.

In figure 7c the percentage of metabolic nets with all its dissipative elements in an *on-off* switching state is shown. For δ fixed, the percentage increases when the number of subsystems is between 2 and 4, and then it decreases, tending to 0 asymptotically with the number of subsystems. The global maximum (30.9%) is reached for δ = 0.9 and *n = 3*, and the global minimum (0.11%) is reached for δ = 0.1 and *n = 50*.

In figure 7d the percentage of DMNs that develop a metabolic core of subsystems locked into an active state while the rest of metabolic subsystems exhibit an *on-off* changeable state is shown. When δ is fixed, the percentage grows pronouncedly with the number of subsystems. The rank of growth (that is, the difference of the percentage between *n = 50* and *n = 2*) is 59.3 for δ = 0.1 and 76.3 for δ = 0.9. For a given *n* with *n>35* the percentage increases notably with δ.

The minimum percentage (0.61%) is attained when δ = 0.1 and *n = 2*, and the maximum (77.71%) is obtained when δ = 0.9 and *n = 50*.

It can be observed that an increase in the number of subsystems that constitute the net results in a decrease in the percentage of nets functionally non-viable (with all their subsystems *off*) as well as the nets with all their subsystems unable to change state and the nets with all their subsystems changing. Nevertheless, this increment in the number of subsystems suggests an asymptotic trend to reach a percentage of 100% of the nets presenting a global configuration characterized by having a metabolic core of subsystems locked into an active state while the rest of metabolic subsystems exhibit an *on-off* switching state.

These data seem to indicate that the fundamental element for the emergence of a global functional configuration characterized by presenting a metabolic core is a high number of metabolic subsystems.

## Discussion

In order to study the emergent behaviors in metabolic structures formed by dissipative enzymatic associations (metabolic subsystems) connected by substrate fluxes and regulatory signals (allosteric and covalent interactions) we have used a type of dynamic system, which we call “dissipative metabolic networks” or DMNs.

Three types of basic emergent behaviors can be distinguished:

- Dynamic transitions corresponding to the mean amplitude, amplitude and frequency in the activities of each subsystem.
- Activity patterns developed by the metabolic subsystems.
- Global functional configurations in the network.

Upon the numerical analysis, a number of different qualitative types of transitions among the activity patterns of the subsystems can be observed: steady state-steady state, steady state-periodic regime, changes between different periodic regimes and chaotic transitions.

Periodic and chaotic transitions in these activity patterns of the subsystems are common. For the case of periodic cycles of transitions, the subsystems run repeatedly through the same set of states, resulting in a cycle of distinct periodic oscillations and steady states. Chaotic behaviors can be observed even in very simple nets of two or more subsystems with a single flux and a unique regulatory signal sent by a catalytic element in the network.

Each metabolic subsystem may present one of the three general states: always *on*, always *off*, or an *on-off* changing dynamics. If we consider the dynamic behaviors of a metabolic network regarding only these three general states, interesting global functional configurations in the overall of the net can be observed.

In fact, for a specific parameter value (for example, the threshold value in the regulatory signals of total inhibition, i.e. the level of enzymatic covalent activity) all the subsystems can be in an *on* state. In this situation, the dynamics are restricted to the possible changes in the variables of the active subsystems.

Further modifications in the control parameter may lead to a new global configuration in which all their dissipative subsystems are in an *on-off* changeable state.

A new variation in any control parameter may provoke a qualitative change in the metabolic net, resulting in the emergence of a new global configuration, in which a certain number of subsystems are locked in an active state (metabolic core) while the rest of the subsystems remain in an *on-off* changing dynamics. Besides, all these metabolic subsystems, both those locked in an always active state and those which are permanently in a process of activation and inhibition, exhibit local dynamics with transitions between different steady states and oscillatory behaviors.

In the prevailing conditions inside the cell, catalytic dynamics seem to show a similar structure to this kind of metabolic global configuration. The cellular metabolism presents a metabolic core formed by a set of catalytic associations always in active states (in eukaryotic cells they are mainly the tricarboxylic acid cycle, the pyruvate dehydrogenase complex and the oxidative phosphorylation) while the rest of the catalytic subsystems are in an *on-off* changing state (β-oxidation of fatty acids, amino acid degradations, glycolysis, gluconeogenesis, etc).

Since this global metabolic dynamic may be a genuine and universal functional configuration of the cellular metabolic structure common to all living cells, we have focused our efforts on analyzing the elements which may determine its emergence.

With this purpose, in a first study, we have analyzed 1.210.000 different randomly constructed metabolic nets with only 12 subsystems and with three regulatory signals and two input fluxes subsystem.

The numerical results show that more than 50% of the analyzed nets present a global configuration characterized by exhibiting a metabolic core.

Regarding the rest of the global metabolic configurations, apart from appearing in smaller percentages than the previous one, it is observed that these percentages diminish even more when the level of covalent regulation is not very high, as could be expected in living cell conditions (here small threshold values δ represent high covalent regulatory activity).

In fact, when the nets with all their subsystems unable to change its state are studied (this happens for the 33.26% of the networks), it is noted that the smallest number of these nets is obtained when the level of covalent regulations is intermediate and the influence of the past is high (but not maximum, that is β<1).

In the particular case of nets with all their subsystems off (that case holds for the 4.95% of the nets, and constitutes part of the 33.26% of the nets unable to change its state), it can also be observed that the percentage of such nets is less than 1% when the level of covalent regulation is intermediate or low and the influence of the past is not very high (β<0.7).

As in the previous cases, when nets in which all the subsystems are in an on-off changeable state are considered (this happens for the 14.09% of the nets), it is observed that the lowest percentages are obtained when the level of covalent regulation is not high and the influence of the past is low.

The nets characterized by having a metabolic core, appear in the highest percentages, which become particularly high (up to 80%) when the level of covalent regulations is not high and β is low.

In the light of these results, despite the fact that the control parameters β and δ may cause important changes in the dynamic behaviors of the nets, it seems clear that none of these control parameters are determinant in the emergence of a global metabolic configuration characterized by a set of subsystems locked into an active regime while the rest is in an *on-off* switching state in any generic metabolic net.

In order to better understand which elements could determine the emergence of this global configuration, we have carried out simulations including 1.250.000 randomly constructed nets, using the number *n* of subsystems that conform each network and the threshold value δ (the enzymatic covalent regulatory activity level) as control parameters.

The obtained results seem to show that the percentage of nets with all their subsystems unable to change the state decreases remarkably with increased numbers of subsystems. This decrement is more notable when the level of covalent regulation in these nets is not high.

In the particular case of nets with all their subsystems in an *off* state (functionally unviable metabolic nets) their percentage tends asymptotically to zero. In fact, when the number of subsystems is 50, the percentage is negligible.

An analysis of the nets with all their subsystems in an on-off changeable state shows clearly that this percentage tends also to zero.

Finally, the study of the remaining nets (the ones with a metabolic core) shows a rapid increase in their percentage as a function of the number of subsystems. These values are particularly high when the level of covalent regulation is moderate. These results suggest that there is an asymptotic trend leading towards 100% in the percentages of this global functional configuration when the number of subsystems increases.

In conclusion, the fundamental element for the emergence of a global metabolic configuration characterized by presenting a metabolic core is the number of subsystems. It seems that this global configuration is an emergent property of all the DMNs with a high number of subsystems.

It is possible that the complexity inherent to a biochemical system conformed by a high number of dissipative subsystems provokes the spontaneous emergence of this fundamental aspect of the cellular metabolic structure.

Unicellular organisms present a kind of metabolic cellular structure characterized by having a dynamic functional configuration in which a small group of metabolic subsystems are always active whereas the rest present on-off catalytic dynamics. Our numeric analysis seems to show that this global dynamic self-organization of the metabolism emerges spontaneously in the cellular metabolic space, as a consequence of the fact that the cells possess catalytic structures conformed by a big number of metabolic subsystems.

All living cellular organisms seem to display some metabolic subsystems that are always active, (these are involved in the permanent production of ATP), whereas the rest of the subsystems present on-off reactive dynamics. Taking all this into account, we can conclude that this global dynamic organization of the reactive cellular processes is possibly a genuine characteristic of the metabolism, common to all living cellular organisms, which could be basic and fundamental in the regulation of the most elementary cellular processes.

By means of modelling approaches, such as the constraint-based analysis applied to metabolic networks, E. Almaas, A.L. Barabási and their group of researchers, [12], [64] have shown that the global organization of metabolic fluxes in certain bacterial cells is characterized by displaying a metabolic core of catalytic reactions always active under different growth conditions, embedded in a connected set of enzymatic reactions where some enzymatic pathways are eventually turned off completely.

These studies have been made in Escherichia coli, Helicobacter pylori, and Saccharomyces cerevisiae metabolism, showing also that most current antibiotics may interfere with the metabolic core [64]. The authors suggest that this global organization of the cellular metabolism “probably represents a universal feature of metabolic activity in all cells, with potential implications for metabolic engineering.”

The global configuration that emerges in the DMNs, characterized by displaying a metabolic core formed by a set of subsystems always active embedded in a set of subsystems in an on-off changeable state, seems to agree remarkably with the observations of E. Almaas, A.L. Barabási and their group of researchers. Our numeric results seem to show that this metabolic global organization is originated by the dissipative dynamics that spontaneously emerge in the cellular reactive structure formed by a network with a high number of enzymatic processes.

On the other hand, the emergence of frozen cores in dynamic networks has also been pointed out in Random Boolean networks. This interesting kind of dynamical systems was introduced in 1969 by S. Kauffman [81]–[83].

Under experimental conditions, most of the local dynamic behaviors of the metabolic subsystems exhibit periodical oscillatory patterns or steady states. However, it has also been observed that some subsystems can present chaotic activities. For example, that occurs in oscillations in citric acid cycle (an always active metabolic subsystem) and in calcium oscillations in oocytes where the membrane receptors are activated.

Our results also show that the mean amplitude, amplitude and frequency in the activities of certain subsystems may present chaotic patterns. These dynamic behaviors may emerge in the simplest nets with only two subsystems and in the most complex nets; likewise, in subsystems always *on* as well as in metabolic subsystems that exhibit an *on-off* switching state chaos can be observed.

The combination of chaotic dynamics in some subsystems with a stable functional configuration which presents a metabolic core may be of biological interest. The stability of this global configuration is necessary to ensure the maintenance of its metabolic structure. The existence of chaotic dynamics in the transitions of the subsystems activity may constitute an advantage in the self-regulatory control of the system, due to the sensitivity of chaotic behaviors to initial conditions; these dissipative metabolic networks with local chaotic patterns may permit fast responses during the cellular adaptation and in the regulation against perturbances.

This conception of the cellular metabolic structure as a complex dissipative catalytic network endowed with a stable global configuration in which a core of metabolic subsystems are locked into an active regime, while the rest present complex *on-off* changing dynamics, and able to simultaneously develop steady states, regular oscillations and chaotic transitions, may help to better understand cytological phenomena and to reinterpret them in a closer to reality way.

## Materials and Methods

### 1. Dissipative Metabolic Networks model

DMNs consist of a set with *n* interconnected elements, called metabolic subsystems or MSbs. Each subsystem represents a group of enzymes aggregated in clusters and dissipatively structured. This enzymatic set is considered as an individual catalytic entity.

The subsystems receive both input fluxes (the substrates of the biochemical reactions) and regulatory signals, which may be of three types: activatory, inhibitory and total inhibitory (all-or nothing type). These interactions correspond with the activity of allosteric enzymes and regulatory enzymes of covalent modulation.

Each MSb converts the input fluxes and regulatory signals into a unique or several output activities and sends one or several fluxes and regulatory signals to other subsystems.

The output activity can be periodic or stationary (In accordance with the experimental observations, in which most of the patterns are stationary or correspond with periodic oscillatory behaviors [33]).

The conversion from input to output activity is performed in two stages. In the first one, the input fluxes are transformed in an intermediary activity using some of the “flux integration functions”. In the second stage, the received regulatory signals originate a “regulatory signal integration” which varies the intermediary activity.

The magnitude of the influence of each regulatory signal is defined by an associated regulatory coefficient.

In order to simplify the assumptions, we will take first the periodic oscillations to be harmonic; these harmonic oscillations will be interspected later with transition regimes that are combinations of the harmonic oscillations preceding and following the transition. This will result in nonlinear oscillations with different levels of complexity (see, figure 6d), as could be expected in the cellular conditions “in vivo”.

So the activity of the *i-th* subsystem will take the form *y_i_*(*t*) = (*A*_0_)*_i_*+*A_i_*sin(*ω_i_*t), where (*A*_0_)*_i_* is the mean amplitude, *A_i_* is the amplitude of the oscillation and *ω_i_* is the frequency. Since *y_i_*(*t*) has to be non-negative, we will take (*A*_0_)*_i_*≥0 and 0≤*A_i_*≤(*A*_0_)*_i_*. We will also suppose that the values of (*A*_0_)*_i_* and *ω_i_* are bounded, that is, there exist constants (*A*_0_)*_max_* and *ω_max_* such that (*A*_0_)*_i_*≤(*A*_0_)*_max_* and *ω_i_*≤*ω_max_* for all *i*. The function *y_i_*(*t*) can be characterized by three variables, *x_i_*_,1_, *x_i_*_,2_ and *x_i_*_,3_, with values between 0 and 1, by taking (*A*_0_)*_i_* = *x_i_*_,1_(*A*_0_)_max_, *A_i_* = *x_i_*_,2_(*A*_0_)*_i_* and *ω_i_* = *x_i_*_,3_ *ω*_max_ (in fact, the variables *x_i,j_* may be considered as parameters, because they change very slowly in time). Hereinafter, we will identify the activity of the *i-th* subsystem during the *k* – *th* dynamical regime with the triple 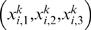, and the activity of the complete set of subsystems with the array 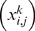, where *i* varies from 1 to *n*, being *n* the number of subsystems and where *j* varies between 1 and 3; the superindex *k* indicates the current iteration.

The activity will be steady when *x_i_*_,2_ = 0 or *x_i_*_,3_ = 0, and the subsystem will be in an inactive state when *x_i_*_,1_ = 0.

The normalized activity of each MSb is 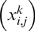.

The temporal period during which each oscillation is maintained will be a constant value that we will call *T*. During the *k-th* iteration the time *t* will vary between *(k-1)T* and *kT*.

We proceed now to describe the way the subsystems are interconnected and how the conversion of the input activities into a unique output activity is realized. As we said above, the activity of the whole DMN in the *k-th* iteration will be
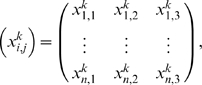
where the index *j* in 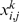 define *A_0_*, *A* and *ω* when *j* is 1, 2 and 3, respectively. This matrix describes the state of the whole net in each iteration and we will call it the state matrix. The integration process that transforms *x^k^* in *x^k^* ^+1^ consists of two stages that will be fully described in which follows. The first stage is the flux integration, in which 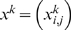 is transformed in an intermediary activity 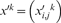 by means of three integration functions: *F*_1_,*F*_2_ and *F*_3_. The second one is the regulatory signal integration stage, in which we pass from 
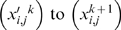
 by using three signal regulatory functions: the activation function AC, the inhibition function IN, and the total inhibition function TI.

### 2. Flux integration

Each subsystem receives an input flux from a subset of the remaining ones. In the simplest case, the *i-th* subsystem receives flux from only one subsystem, say the *l-th*. The input flux will consist of the three values 
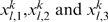
, and the integrated input flux will consist of the numbers 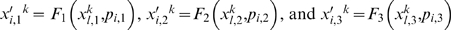.

**Figure 8:**
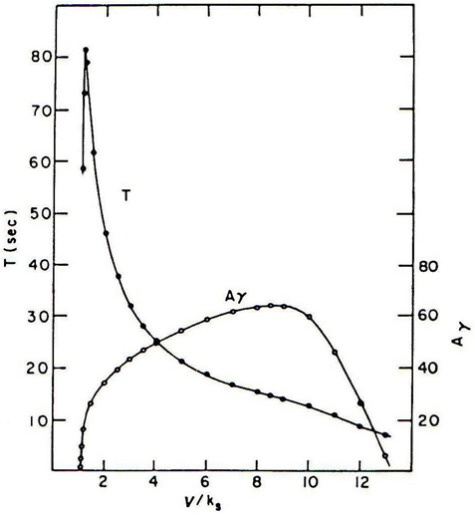
Amplitude and frequency in the glycolytic subsystem. Variation of the period and the amplitude of the oscillations in the glycolitic subsystem in function of the input speed in the substrate made by Goldbeter and Lefever.

The qualitative behaviors of the amplitude and frequency in the glycolytic subsystem were studied in detail by Goldbeter and Lefever [80] (see figure 8). We have used piecewise linear functions approximating the nonlinear functions obtained by Goldbetter and Lefever. Thus, we have calculated the integrated input flux by using the functions *F*_1_,*F*_2_,*F*_3_ defined as follows:
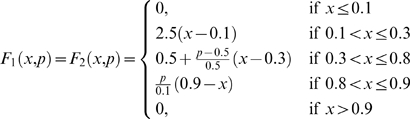
and
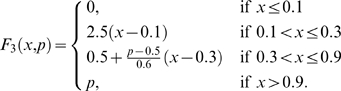
Where *p* is a parameter associated to each flux integration function.

In the most general case, the *i-th* subsystem receives flux from more than one subsystem. We will define the integrated flux as the arithmetic mean of the received integrated fluxes.

Each subsystem can also have an outer input flux, consisting of three fixed values 
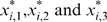
 that are integrated with the same functions *F*_1_,*F*_2_,*F*_3_ that were defined before, but for other parameter values 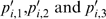. These integrated outer fluxes must be taken into account in the evaluation of the arithmetic mean together with the integrated inner fluxes.

### 3. Signal-regulatory integration

When the flux integration has been done, we proceed next with the signal regulatory integration. First, we will describe the process in the simple case in which a MSb has only one regulatory signal. Let us suppose that the *i-th* MSb receives a regulatory signal from the *l-th* one. Let 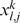 be the *j-th* component (which describe *A*_0_, *A* or ω) of the activity of the subsystem which regulates at the beginning of the *k+1-th* iteration, and 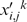 the *j-th* component of the activity of the regulated MSb after the input flux integration.

If the signal is of inhibitory type (negative allosteric modulation), then the activity 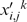 is reduced by multiplying it by a factor 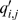 in the unit interval [0,1]. It will be 1 when 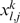 is 0, and a parameter *q_i,j_* when 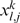 is 1. In the remaining cases it will vary affinely with 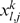, so that 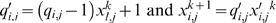. Let us stress that when 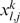 is 0, 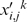 is unaltered, and when 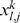 is 1, the inhibition is maximum and the activity is reduced by a factor of *q_i,j_*. Thus, we have 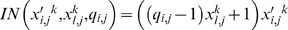.

If the signal is of activatory type (positive allosteric modulation), the function is similar to the one described above, once we take into account that to augment a value *x* in [0, 1] is equivalent to reducing *1-x* to a smaller value *x*′ in [0, 1]. So, we will define the activatory functions by
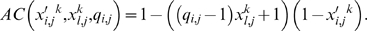



We will call the parameters *q_i,j_* “influence coefficients”; they represent the influence of each modulator on each subsystem.

If the signal is of total inhibition type, then we will use a parameter δ, that will be called threshold. The activity 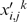 will be unaltered when the activity 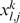 does not reach that threshold and will be 0 when it trespass this value, that is,
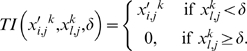



In the general case in which a MSb receives regulatory signals from several subsystems, they act sequentially, so that if there are *r* regulatory signals for each MSb, then we have *r* intermediary states *x^s,k^*, where *s* varies between 1 and *r*, (besides of the intermediary state *x*′*^k^* obtained after the flux integration was done). In this case, we replace in the previous formulas 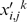, by 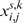 so that if the signal is inhibitory then we have
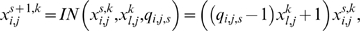
and if the signal is activatory then we have
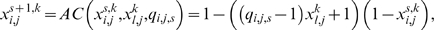
and, finally, if the signal is of the total inhibition type (enzymatic covalent regulation) then we have
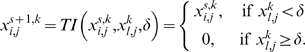



At the end we obtain *x^r,k^* = *x^k^* ^+1^.

Now we will describe some additional rules for the integration of the activities of each subsystem.

When after the flux integration stage 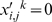 (that is, if the input flux is 0), then we will ignore the signal regulatory integration stage and we put simply 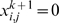. Also, if in the *l-th* stage of the signal regulatory integration a total inhibition acts and the threshold is reached then we will ignore the subsequent regulatory signals for that component (*A_0_*, *A or ω*) and we will take 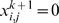. Also, we will ignore regulatory signals from an inactive MSb: if the *i-th* subsystem receives a regulatory signal from the *l-th* one and 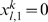, then the corresponding regulatory integration will not be performed.

Finally, when 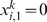, the MSb will be *off* independently of 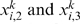. In this case, we will take 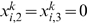.

The DMNs are discrete and deterministic dynamical systems. Once the parameters of the net are fixed, there is only one solution in the net. In the simplest case, each metabolic subsystem presents a unique dynamic behavior, which can be a steady state or a periodic oscillation. However, the net can self-organize spontaneously in such a way that each metabolic subsystem shows a solution characterised by presenting uninterrupted transitions between various behaviors periodic and/or stationary. In the most complex cases deterministic chaotic solutions can emerge spontaneously, originating infinite transitions between different periodic and/or stationary behaviors. In this situation, each metabolic subsystem modifies uninterruptedly its enzymatic activity in such a way that it changes between different periodic and/or stationary regimes that never repeat its activity along arbitrarily long periods of time.

The dynamic behaviors that emerge spontaneously in the DMNs have their origin in the regulatory structure of the feedback loops, in the non-linearity of the constitutive equations of the system and in the complexity of the own dynamics that are generated in the network.

### 4. Representation of the activity of the metabolic subsystems

Now we will describe the way we pass from a sequence of terns 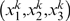 representing the mean amplitude, amplitude and angular speed of a given subsystem to a continuous function representing the activity level of the subsystem. We consider a number *N* of transitions and, in the *k-th* stage, we suppose that the oscillation is harmonic, that is, the activity of the subsystem is described by a function of the form *y*(*t*) = (*A*_0_)+*A* sin(*ω*t), where 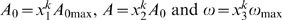 and where *A*_0max_ and *ω*_max_ are given fixed parameters independent of the stage number and of the subsystem. The duration of the harmonic oscillation is a given parameter *T_h_* independent also of the stage and of the subsystem. In between two stages, a mixed transition regime is maintained with a duration *T_tr_* independent of the stage number and of the subsystem. If the transition goes from the *k-th* stage to the *(k+1)-th* stage then, during the *T_tr_* seconds of transition regime, the activity is given by a function of the form *y*(*t*) = *A*(*t*)*y*_1_(*t*)+*B*(*t*)*y*_2_(*t*), where *y*_1_(*t*) is the activity corresponding to the prolongation in time of the previous harmonic activity in the *k-th* stage, and *y*_2_(*t*) is the back-propagation in time of the subsequent harmonic activity in the *(k+1)-th* stage. The numbers *A*(*t*) and *B*(*t*) depend on time and indicate the weights with which the activities of the subsystem in the previous and posterior stage are present during the transition time. At the beginning of the transition, say at *t* = *t*_0_, *A*(*t*_0_) is 1 and *B*(*t*_0_) is 0, and at the end of the transition, say at *t* = *t*_1_, *A*(*t*_1_) is 0 and *B*(*t*_1_) is 1. At the rest of the transition times *A*(*t*) and *B*(*t*) vary affinely. Thus, 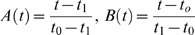. Putting all this together, during the transition time the activity is given by
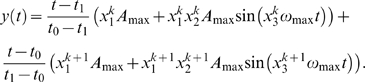



The transition regimes are combinations of two harmonic oscillations with nonconstant coefficients *A*(*t*) and *B*(*t*) depending on time. Thus, the introduction of these transition regimes provokes the emergence of nonlinear oscillatory behaviors, both simple and complex (see for example figure 6d).

### 5. Dependence of the past

In an attempt to take into account a influence of the past in some of the DMNs, taking into account that each activity of the subsystem (besides of depending on incoming fluxes and regulatory signals) depends also on some activities developed in the past, we have considered the following aspects: the state 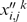 resulting from the flux integration and signal-regulatory-integration stages is augmented or diminished in function of 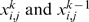 (i.e., of the state in the *k-th* stage and of the previous state); when 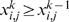, 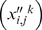 is diminished, and when 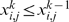, 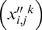 is augmented. In the first case, 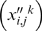 is multiplied by a factor *β*′ belonging to [0, 1] whose value is 1 when 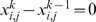, a fixed constant β when 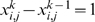 and have an affine variation with 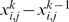. Thus
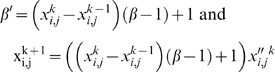



In the second case, the difference 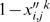 is reduced multiplying it by an analogous factor *β*′ belonging to [0, 1] whose value is 1 when 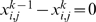, the same constant β of the precedent discussion when 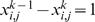 and have an affine variation with 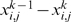.

Thus 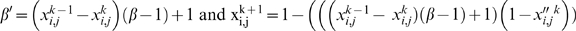. Then,
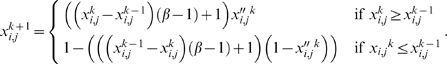



To overcome the need to know two initial states in the first stage, that is, when *k* = 1, we have obtained the state *x* ^2^ from *x* ^1^ without taking into account the dependence of the past, which is equivalent to define *x* ^0^ = *x* ^1^.

In some of our studies we have considered β as one of the control parameters.

### Example 1

We will consider the simple DMN formed by two subsystems arranged in series with two feedback loops of regulatory signals. The MSb1 is activated by the second subsystem and the MSb2 is totally inhibited by the first subsystem when this one reaches a determinate threshold value (figure 1). The MSb1 input flux value is 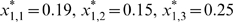, with 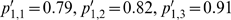. The parameter values for the integration functions of MSb2 are: *p*_2,1_ = 0.78, *p*_2,2_ = 0.83, *p*_2,3_ = 0.94. The catalytic dissipative element MSb1 is activated by the second MSb, with *q*_1,1_ = 0.18, *q*_1,2_ = 0.02, *q*_1,3_ = 0.15 and the MSb2 is totally inhibited by MSb1, with a threshold δ = 0.1. We have fixed the value β = 0.7, where β is the past influence coefficient.

The initial state is
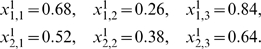



As we said before, we will not consider the influence of the past for the calculation of the next state, and we will use only the flux integration and the regulatory integration. The details of these integrations will be omitted for brevity in this first stage, but the calculations will be completely developed in the next stage. The second state that we obtain is
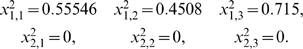



After the flux integration stage we reach an intermediary state
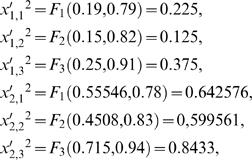



After the signal regulatory integration stage we obtain the following state
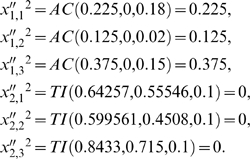
And, considering the influence of the past for β = 0.7 we obtain
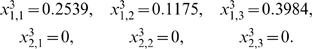



Finally, in the DMN the first MSb will fall into a single active state, corresponding to a periodic oscillation with *A_0_* = 0.225, *A* = 0.125, *ω* = 0.35, and the second MSb is locked into an inactive state.

### Example 2

**Figure 9:**
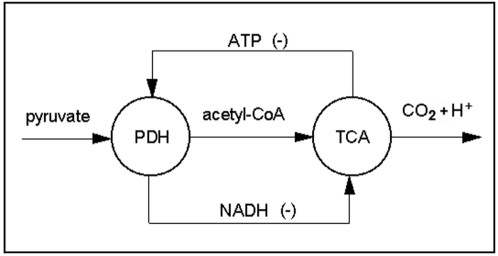
Network with two metabolic subsystems: pyruvate dehydrogenase and Krebs cycle. DMN formed by two subsystems arranged in series, which represent in a very simplified way the main interconnections between the pyruvate dehydrogenase complex (PDH) and Krebs cycle.

Next, we have considered a DMN formed by two subsystems arranged in series, which represent in a very simplified way the main interconnections between the pyruvate dehydrogenase complex (PDH) and Krebs cycle, so-called tricarboxylic acid cycle (TCA) (Fig. 9).

Pyruvate dehydrogenase is a large multienzymatic subsystem containing many copies of three enzymes: pyruvate dehydrogenase, dihydrolipoyl transacetylase and dihydrolipoyl dehydrogenase. This metabolic subsystem (MSb1) receives an input flux of pyruvate yielding acetyl-CoA, H^+^ and NADH by a process called pyruvate decarboxylation which is mainly inhibited by the ratio ATP/ADP [62]. When ATP, the energy-rich end product of the tricarboxylic acid cycle and oxidative phosphorylation, accumulates to high levels, the rate of formation of acetyl-CoA is slow.

Acetyl CoA, a product of the pyruvate dehydrogenase reactions is a central compound in metabolism with several functions: as input to the Krebs cycle, where the acetate moiety is further degraded to CO2, and as donor of acetate for synthesis of fatty acids, ketone bodies, and cholesterol.

One of the factors controlling the MSb2 is the NADH. The citrate synthase enzyme is the primary control point in the Krebs cycle and is negatively regulated by NADH. Isocitrate dehydrogenase and α-ketoglutarate dehidrogenase are also strongly inhibited by the negative allosteric modulator NADH [62].

Therefore, the catalytic dissipative element MSb1 is inhibited by the ATP from the second subsystem and the MSb2 receives an inhibitory signal of NADH from the MSb1.

The parameters considered in this DMN are the following ones: the first subsystem MSb1 receives an input flux of pyruvate with 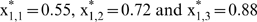, being the values of the parameters of the integration functions of (p_1,1_ = 0.54, p_1,2_ = 0.60, p_1,3_ = 0.78) and β = 0.2. The initial states are the same that the ones belonging to the DMN shown in the example 1.

The MSb2 receives an input flux of acetyl-CoA from MSb1, being the values of the parameters of the integration functions of (p_2,1_ = 0.398, p_2,2_ = 0.516, p_2,3_ = 0.5).

In order to simplify we have equalized the three influence coefficients of A_0_, A and ω belonging to each inhibitory signal (*q_i_*_1_ = *q_i_*_2_ = *q_i_*_3_ = *q_i_*).

First, we have considered as control parameter *q_1_*, the ATP influence coefficient, and we have fixed *q_2_* = 0.01 which represents the influence coefficient value of NADH (small values of the influence coefficient represents high inhibitory activity so for *q_1_* = 0.1 and *q_2_* = 0.01 both subsystems are strongly inhibited).

After the numeric integration of the DMN it can be observed that for 0.1≤*q_1_*≤0.9 both subsystems are active and the two subsystems exhibit one periodical activity. Some of the values of the amplitude and oscillatory frequencies are shown below:

*q_1_* = 0.1 (MSb1) A_0_ = 0.3085, A = 0.3614 and ω = 0.3457.
   (MSb2) A_0_ = 0.3477, A = 0.3290 and ω = 0.3429.
*q_1_* = 0.5 (MSb1) A_0_ = 0.3779, A = 0.4366 and ω = 0.4187.
   (MSb2) A_0_ = 0.3168, A = 0.2994 and ω = 0.3252.
*q_1_* = 0.9 (MSb1) A_0_ = 0.4360, A = 0.4994 and ω = 0.4847.
   (MSb2) A_0_ = 0.2904, A = 0.2729 and ω = 0.3049.

When the inhibition provoked by the ATP descends (by increasing the values of the influence coefficient) the activity of the MSb1 increases as well as the contribution of NADH and as a consequence of it the activity of the MSb2 descends.

We have considered now *p_2_* as control parameter, the NADH influence coefficient, and we have fixed the ATP influence coefficient value (*q_1_* = 0.01). This *q_1_* value represents a strong inhibition of the pyruvate dehydrogenase complex due to high levels of the energy-rich end product of the tricarboxylic acid cycle and oxidative phosphorylation. Under these parametric conditions the metabolic network can present different dynamic behaviors for *q_2_* values.

When *q_2_* ranges from 0.1 to 0.87, both subsystems are active and exhibit a single periodical activity. For example:

*q_2_* = 0.1 (MSb1) A_0_ = 0.2918, A = 0.3331 and ω = 0.3205.
   (MSb2) A_0_ = 0.3536, A = 0.3547 and ω = 0.3626.
*q_2_* = 0.5 (MSb1) A_0_ = 0.2783, A = 0.2987 and ω = 0.2946.
   (MSb2) A_0_ = 0.3838, A = 0.4225 and ω = 0.4119.
*q_2_* = 0.87 (MSb1) A_0_ = 0.2684, A = 0.2859 and ω = 0.2825.
    (MSb2) A_0_ = 0.4063, A = 0.4476 and ω = 0.4394.

As it can be observed, for *q_2_* = 0.1 the two subsystems are strongly inhibited and both show a low activity. When increasing *p_2_* values (0.1<δ≤0.87) the inhibition provoked by the NADH decreases and as a consequence of it the activity of the MSb2 is increased and the MSb1 activity diminishes due to the increment in the production of ATP.

For 0.87<*q_2_*≤1 the two subsystems undergoes a variation of the oscillatory evolution, emerging very complex transitions and the output activity of the both subsystems make uninterrupted transitions between more of 200 different kinds of periodic oscillations.

Under experimental conditions, the complex temporal structure in Krebs cycle has been observed [78] and it has been verified the complex transitions that characterize the intermediates of the Krebs cycle. See figure 5a for more details.

In the network the two metabolic subsystems remain always active for all the parametric conditions studied.

### 6. Experimental calcium oscillations in *Xenopus laevis* oocyte

In order to study chaotic oscillations in a subsystem with an *on-off* changeable regime we carried out experimental observations of intracellular calcium oscillations in *Xenopus laevis* oocyte.

Laboratory-reared *Xenopus laevis* frogs were obtained from Blades Biological (Cowden, Kent, UK). Oocytes at stageV were plucked from the ovaries and defolliculated by treatment with collagenase (type 1, Sigma) at 80–630 units/ml in frog Ringer's solution (115 mM NaCl, 2 mM KCl, 1.8 mM CaCl, 5 mM HEPES at pH 7.0) for 20 min to remove the surrounding follicular and epithelial cell layers. Oocytes were maintained at 18°C in Barth's solution (88 mM NaCl, 1 mM KCl, 2.4 mM NaHCO_3_, 0.33 mM Ca(NO_3_)_2_, 0.41 mM CaCl_2_, 0.82 mM MgSO_4_, 5 mM HEPES at pH 7.4+gentamicin 70 µg/ml).

Membrane currents were recorded with a standard two-electrode voltage clamp (Warner Instruments, Oocyte Clamp OC-725C) and digitized in a PC computer (Digidata 1200 and Axoscope 8.0 software, Axon Instruments). Oocytes were continually superfused with Ringer's solution at room temperature (∼22°C).

The membrane was usually voltage clamped at −60 mV. Fetal Bovine Serum (FBS) (Sigma-Aldrich) diluted 1∶1000 in Ringer solution were perfused to achieve the generation of voltage oscillations.
